# Core Features of the GP to be transferred during JADECARE and transfer challenges

**DOI:** 10.1093/eurpub/ckac129.508

**Published:** 2022-10-25

**Authors:** J Txarramendieta, J Roca, M Zahorka, K Strand Kudajewski

**Affiliations:** 1 Kronikgune Institute for Health Services Rese, Barakaldo, Spain; 2 August Pi i Sunyer Biomedical Research Institute, Barcelona, Spain; 3 Optimedis AG, Hamburg, Germany; 4 Health Innovation Centre of Southern Denmark, Odense, Denmark

## Abstract

In this second presentation, the representatives of each of the four JADECARE oGPs, Jon Txarramendieta (Kronikgune, Basque Country, Spain), Josep Roca (August Pi i Sunyer Biomedical Research Institute, Catalonia, Spain), Manfred Zahorka (Optimedis AG, Germany) and Kuno Strand Kudajewski (Health Innovation Centre, Southern Denmark) will describe the main traits of the Core Features of their practices and the Next adopters that will transfer and adapt them highlighting the transfer challenges.

